# Plasma Proteomic Signatures for Diverticulitis Risk Stratification

**DOI:** 10.1016/j.jss.2025.06.093

**Published:** 2025-08-23

**Authors:** Thomas E. Ueland, John P. Shelley, Jonathan D. Mosley, Jamie R. Robinson, Eric R. Gamazon, Lillias H. Maguire, Richard Peek, Alexander T. Hawkins

**Affiliations:** a Vanderbilt University School of Medicine, Nashville, Tennessee; b Department of Internal Medicine, Vanderbilt University Medical Center, Nashville, Tennessee; c Department of Biomedical Informatics, Vanderbilt University Medical Center, Nashville, Tennessee; d Department of Pediatric Surgery, Vanderbilt University Medical Center, Nashville, Tennessee; e Division of Genetic Medicine, Department of Medicine, Vanderbilt University Medical Center, Vanderbilt Diabetes Research and Training Center, Nashville, Tennessee; f Department of Surgery, University of Pennsylvania, Philadelphia, Pennsylvania; g Division of Gastroenterology, Hepatology, and Nutrition, Vanderbilt University Medical Center, Nashville, Tennessee; h Division of General Surgery, Vanderbilt University Medical Center, Section of Colon & Rectal Surgery, Nashville, Tennessee

**Keywords:** Diverticulitis, Diverticular disease, Phenome-wide association study, Polygenic risk score, Precision medicine, Proteomics

## Abstract

**Introduction:**

Approaches for risk stratification in diverticulitis have emphasized lifestyle factors, with a possible emerging role for molecular signatures. We aimed to evaluate whether plasma proteomic profiles complement dietary and genetic factors in diverticulitis risk stratification.

**Materials and methods:**

This UK Biobank study derived a plasma proteomic risk score for severe diverticulitis (operative or recurrent inpatient disease). The cohort was split into a training set for derivation and an independent testing set for evaluation. Differential expression and gene set enrichment analysis identified pathway-level differences, while least absolute shrinkage and selector operator models calculated the score. To evaluate utility in stratification, the proteomic risk score was included in Cox regression models with demographics, lifestyle factors, and genetic risk. A phenome-wide association study explored for conditions associated with diverticulitis proteomic signatures.

**Results:**

Among 43,539 patients and 1459 plasma proteins measured at enrollment, there were 551 cases of severe diverticulitis throughout follow-up. Differential expression analysis implicated extracellular matrix and neuronal pathways, while least absolute shrinkage and selector operator regression retained 151 proteins. This proteomic risk score was associated with greater risk of severe diverticulitis (hazard ratio [95% confidence interval], 1.48 [1.18–1.87]), and a full model with proteomic and genetic factors improved upon a base model with demographic and lifestyle factors (maximum at 5-y area under the receiver operating characteristics curve [95% confidence interval], training set: 0.83 [0.79–0.86] *versus* 0.69 [0.64–0.73]; testing set 0.75 *versus* 0.70; *P* < 0.01) In the phenome-wide association study, elevated proteomic risk for diverticulitis was associated with renal dysfunction and cardiometabolic traits.

**Conclusions:**

Plasma proteomic profiles complemented genetic and lifestyle factors in diverticulitis risk stratification.

## Introduction

The diverticular disease severity spectrum ranges from asymptomatic diverticulosis to complicated diverticulitis requiring operative attention.^1^ Tools to stratify patients for severe presentations are limited,^2^ and existing approaches have focused on dietary patterns or episode history.^3^ Recent efforts from large biobanks have enabled investigations of molecular signatures.^4–6^ For example, over 100 genetic variants have been associated with diverticular disease, and polygenic risk scores capturing patterns across many genetic variants have shown promise for risk stratification.^7–9^ However, genotypes do not guarantee manifestations of disease, as there is a complex regulatory network that orchestrates expression of downstream cellular components.^10^

The plasma proteome may be a useful adjunct for understanding the molecular underpinnings of diverticulitis. While genes are often conceptualized as the blueprint for disease, proteins act directly in biological pathways and may better reflect contributions from environmental stressors.^11,12^ In addition to relationships between a single biomarker and disease, there are approaches that summarize the effects of many disease-associated proteins.^13^ These multiprotein signatures have been investigated as a possible complement to clinical and genetic factors for individualized risk prediction.^14–18^ It is unknown whether there are meaningful systematic differences in the plasma proteome among patients who develop severe presentations of diverticulitis.

This study aimed to derive a plasma proteomic signature for diverticulitis, evaluating biological pathways and potential value in clinical stratification. We hypothesized that a proteomic signature would reflect dysregulation in connective tissue biology. However, we believed that information from proteomic risk would largely overlap with a polygenic risk score when modeling future disease onset.

## Methods

### Study population, phenotyping, and design

The UK Biobank recruited nearly 500,000 participants between 2006 and 2010, combining health surveys with biological samples and inpatient clinical records.^5^ Protein analytes from cardiometabolic, inflammation, neurology, and oncology panels were measured at the baseline visit for 46,595 participants as part of the UK Biobank Pharma Proteomics Project. Measurement details and quality control steps have been described elsewhere.^19^ Our study included UK Biobank participants with available protein expression data at the baseline visit and no diverticular disease diagnosis codes assigned prior to study enrollment. Proteins with more than 10% missingness in the cohort were removed, and samples with more than 20% missingness across all proteins were excluded. Remaining missing protein values were imputed using the cohort median. We identified cases of incident severe diverticulitis and controls without diverticular disease based on International Classification of Diseases (ICD), 10^th^ revision (ICD-10) codes and 9^th^ revision (ICD-9) codes of K57 and 562, respectively. Severe diverticulitis was defined as a participant who met any of the following criteria: 1) underwent a colectomy or percutaneous drain associated with an inpatient admission where a diverticular disease code was assigned as the primary diagnosis, 2) multiple inpatient admissions associated with diverticular disease, 3) diverticular disease listed as the cause of death. Controls were defined as participants without any diverticular disease diagnosis codes by final follow-up (Supplementary Material). The UK Biobank has been approved by the North West Multicentre Research Ethics Committee and this study’s analysis was granted under UK Biobank application ID 100702.

**Fig. 1 –:**
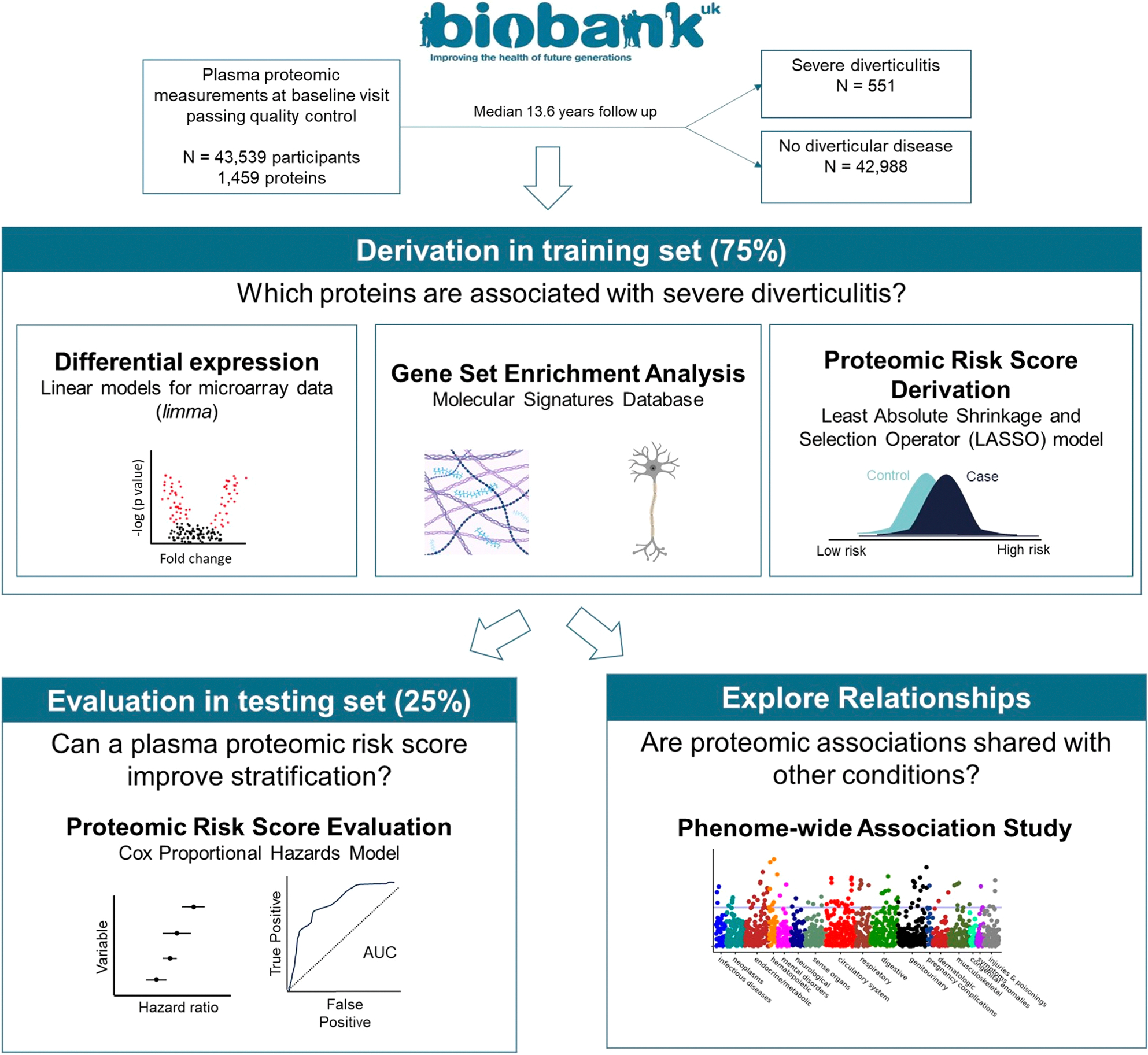
Study design.

The cohort was split into a training set (75%) and a testing set (25%). In the training set, we derived a proteomic risk score and investigated relevant biological pathways from the proteomic perspective. In the testing set, we evaluated the performance of the proteomic risk score in the context of known risk factors for diverticulitis. Finally, we performed a phenome-wide association study (PheWAS) to understand whether a proteomic susceptibility to diverticulitis was shared with other conditions. The study design is summarized in Figure 1.

### Differential expression

We used linear models for microarray data (*limma*) to identify plasma proteins that were differentially expressed in severe diverticulitis.^20^ A linear model was fit with an outcome of the normalized protein expression with covariates of age, sex, and case status (severe diverticulitis *versus* control). Subsequent empirical Bayes moderation via the eBayes function was applied to calibrate perprotein variances using information from all the proteins. Effect sizes were quantified through log_2_ fold change in protein expression with 95% confidence intervals (CIs) between the groups. To account for multiple comparisons, we applied the Benjamini–Hochberg method controlling for a false discovery rate of 0.05.

### Gene set enrichment analysis

Gene set enrichment analysis evaluated pathway-level differences in the plasma proteome of patients with severe diverticulitis relative to controls.^21^ All proteins were ranked by *P* values from *limma* models and assessed for enrichment with annotated gene sets from the Molecular Signatures Database. Specifically, we queried the Reactome Knowledgebase, which categorizes biological interactions into functional pathways such as intermediary metabolism or the innate immune system.^22^ A multilevel Monte-Carlo implementation of gene set enrichment analysis was adopted using the fgsea R package with a minimum of 15 overlapping proteins and 5000 permutations.^23^ Effect sizes were quantified through a normalized enrichment score with significance determined through *P* values with Benjamini–Hochberg adjustments for a false discovery rate of 0.05.

### Proteomic risk score derivation

The proteomic risk score was derived using least absolute shrinkage selection and operator (LASSO) regression models in the training set. The model included all available protein expression values as covariates and adjusted for age and sex with an outcome of severe diverticulitis. LASSO regression shrinks coefficients of unimportant variables to zero and has been previously leveraged for high dimensional proteomic data.^15,17,24^ The lambda hyperparameter was optimized through 5-fold cross validation with selection of the simplest model that maximized area under the receiver operating characteristics curve (AUC). The proteomic risk score was calculated by summing the product of each retained protein with the corresponding LASSO coefficient. Scores were then standardized to a mean of 0 and standard deviation (SD) of 1 in the cohort.

### Proteomic risk score evaluation

The proteomic risk score was evaluated through Cox proportional hazard models. A series of models were fit incorporating demographic, clinical, proteomic, and genetic covariates. The base model included age at recruitment, sex, smoking status, body mass index, and a healthy diet score capturing guideline concordance with patterns for processed meats, fish, fruit, vegetables, and whole grains (Supplementary Material).^25,26^ The genetic model additionally included a polygenic risk score derived from the FinnGen Biobank (Supplementary Material).^6^ The protein model additionally included the proteomic risk score from the current study. The full model included all covariates (Base + Genetic + Protein) with an interaction term between genetic and proteomic risk. Performance was quantified through hazard ratios and 95% confidence intervals as well as time-dependent AUC at 2-y, 5-y, and 10-y of follow-up. Generalizability was assessed through bootstrapped replicates of the training set as well as evaluation in a testing set that was independent from participants used to derive the proteomic risk score. Improvement relative to the base model was quantified through likelihood ratio tests of models that were fit in the testing set.^27^

### PheWAS study

A PheWAS consists of a series of logistic regression models that explores across diagnoses in the electronic medical record for associations with a variable of interest. We performed a PheWAS of the proteomic risk score to evaluate whether diverticular proteomic associations were also shared by other conditions. Source ICD-10 and ICD-9 diagnosis codes were collapsed into phecodes using the PhecodeX map prior to the regression modeling to better represent the disease landscape in meaningful terms.^28^ Models controlled for age and sex with a Bonferroni-adjusted two-sided *P* value significance threshold (*P* < 2.1 × 10^−5^) using the PheWAS R package.^29^ Effect sizes were represented through odds ratios and 95% CIs. All analyses were performed using the DNANexus Research Analysis Platform.

## Results

**Fig. 2 –:**
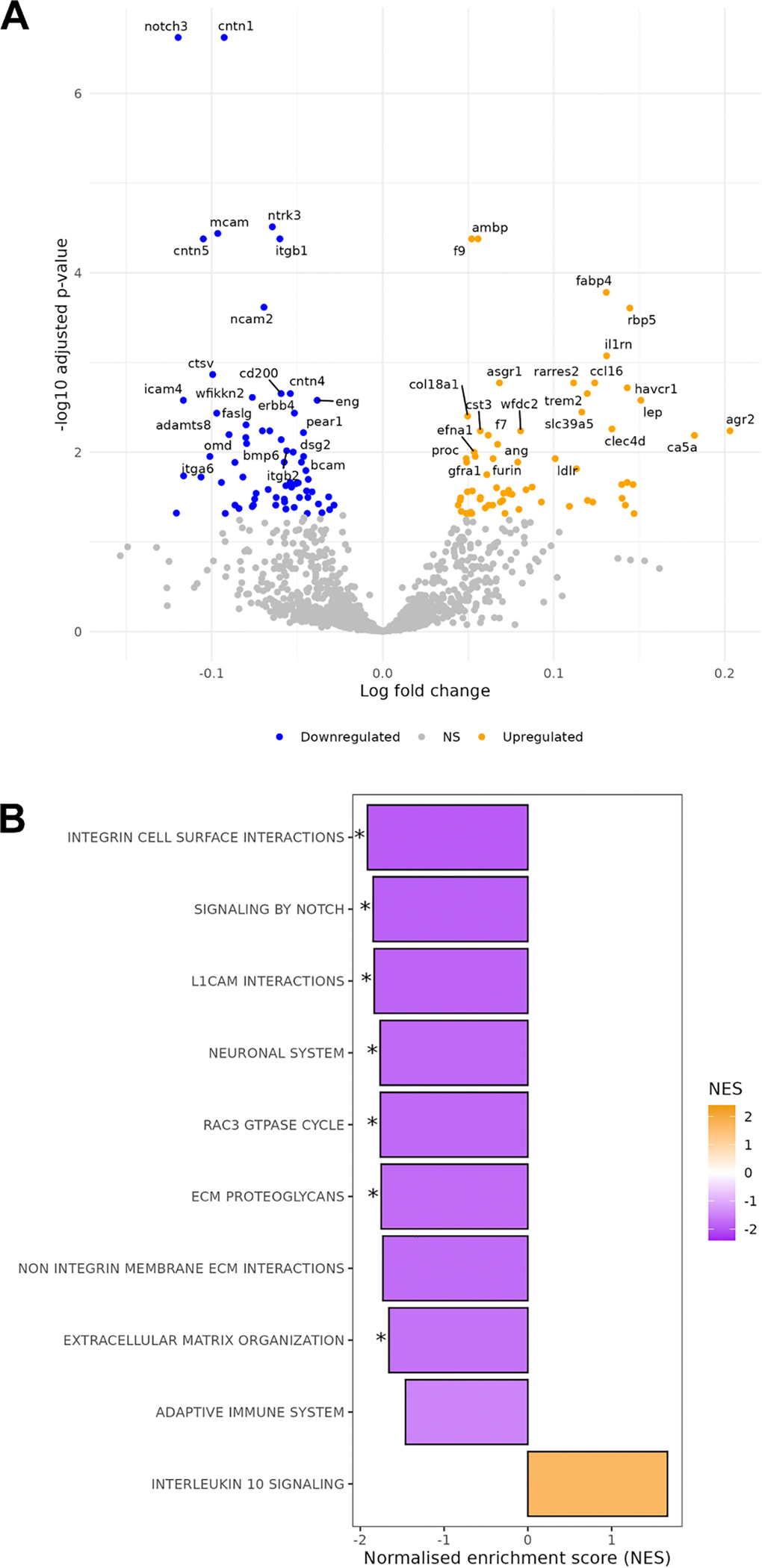
Differential expression and pathways of severe diverticulitis proteins. (A) Candidate proteins identified through linear models for microarray data. Colors represent upregulation or downregulation (B). Gene set enrichment analysis showing normalized enrichment scores among the Reactome gene set from the Molecular Signatures Database. Significance based on *P* values with Benjamini–Hochberg method controlling for a false discovery rate of 0.05. NS: not significant.

There were 43,539 eligible patients with 1459 plasma protein measurements. Over a median (interquartile range) follow-up of 13.6 (1.5) y, there were 551 cases of incident severe diverticulitis (Table 1). Both the training set (75% of cohort) and testing set (25% of cohort) had a mean (SD) age of 56 (8) with 54% female participants. In the differential expression analysis, 66 proteins were downregulated while 65 were upregulated. Reactome pathways corresponding to differentially expressed proteins included “integrin cell surface interactions,” “signaling by Notch,” “L1 cell adhesion molecule interactions,” “neuronal system,” “RAC3 GTPase cycle,” “extracellular matrix proteoglycans,” and “extracellular matrix reorganization” (Fig. 2). The LASSO model included 151 proteins with nonzero coefficients in the proteomic risk score. The ten proteins with the largest weights in the proteomic risk score were cathepsin L2, integrin alpha-6, contactin 1, bone morphogenetic protein 6, SH2 domain–containing protein 1A, ADP-ribosylation factor–binding protein GGA1, coagulation factor IX, neuronal calcium sensor 1, anterior gradient protein two homolog, and triggering receptor expressed on myeloid cells 2 (Supplementary Material).

**Fig. 3 –:**
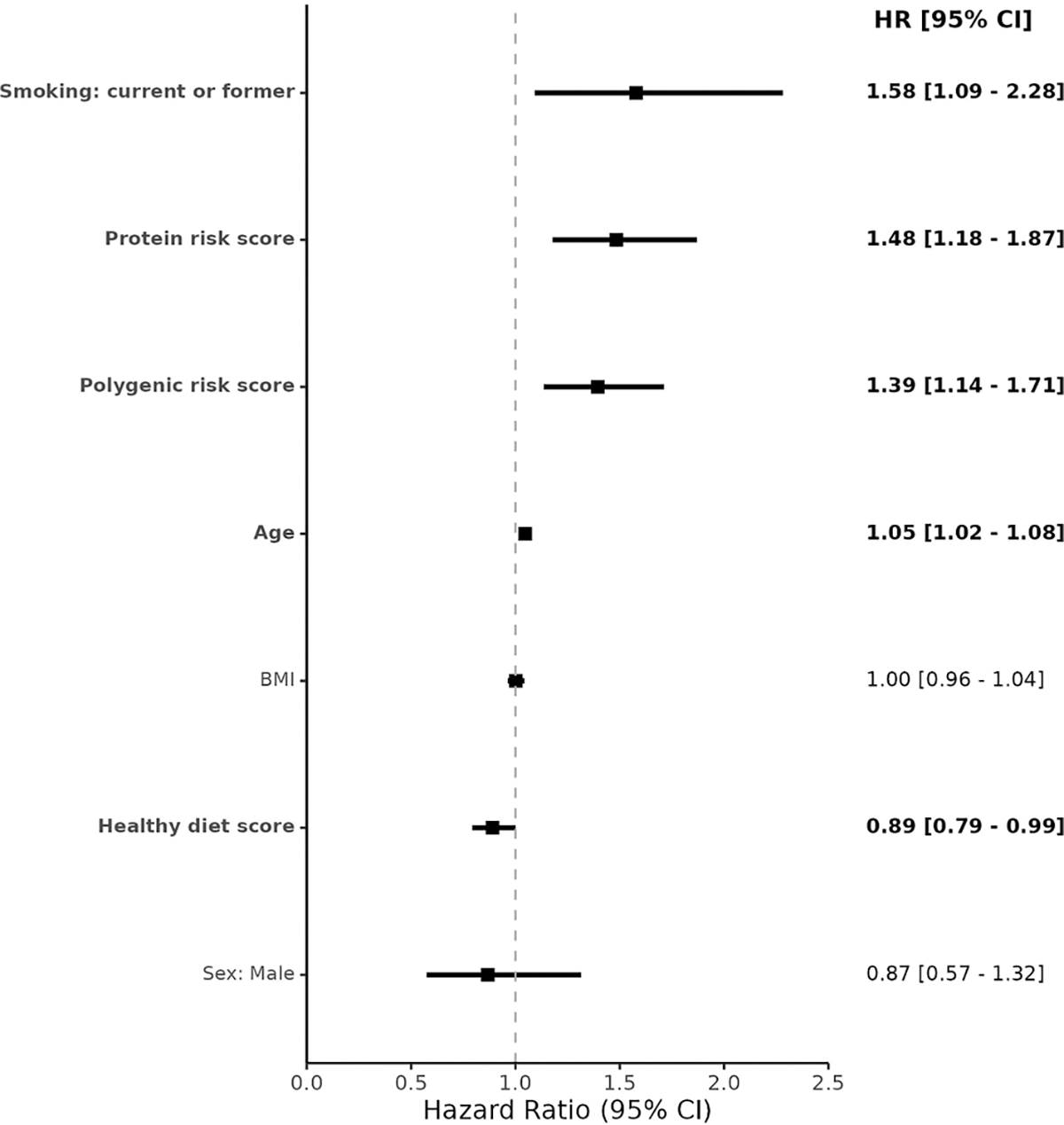
Application phase Cox proportional hazards model in the testing set. Hazard ratios and 95% confidence intervals shown for Cox proportional hazards model fit on the testing set. Model covariates were shown on y-axis, and model outcome was incidence severe diverticulitis. The protein risk score was derived from LASSO coefficients in the training set, while the polygenic risk score was derived from FinnGen summary statistics using PRS-CS. The healthy diet score was derived from self-reported consumption patterns. BMI = body mass index; LASSO = least absolute shrinkage and selection operator.

In Cox proportional hazard models, the proteomic risk score was associated with greater risk of incident severe diverticulitis (HR [95% CI], 1.48 [1.18–1.87]). Additional associations were the polygenic risk score (HR [95% CI], 1.39 [1.14–1.71]), current or former smoking (HR [95% CI], 1.58 [1.09–2.28]), age at recruitment (HR [95% CI], 1.05 [1.02–1.08]), and a healthy diet (HR [95% CI], 0.89 [0.79–0.99]) (Fig. 3). Relative to the base model, discrimination of the full model was higher in the training set at 2 y (AUC [95% CI], 0.84 [0.78–0.89] *versus* 0.72 [0.65–0.78]), 5 y (0.83 [0.79–0.86] *versus* 0.69 [0.64–0.73]), and 10 y of follow-up (0.81 [0.79–0.84] *versus* 0.68 [0.65–0.72]) (Table 2). In the testing set, the base model AUC was 0.71 at 2 y, 0.70 at 5 y, and 0.70 at 10 y, compared to the full model AUC of 0.74 at 2 y, 0.75 at 5 y, and 0.73 at 10 y. Relative to the base model, improvements were observed in the genetic model (likelihood ratio test *P* value: 5.0 × 10^−5^), protein model (likelihood ratio test *P* value: 2.2 × 10^−4^), and full model (likelihood ratio test *P* value: 2.7 × 10^−6^). There was no interactivity between the proteomic and genetic risk terms (*P* = 0.90). The proteomic risk score was poorly correlated with the polygenic risk score (Spearman’s ρ = 0.01) (Supplementary Material).

**Fig. 4 –:**
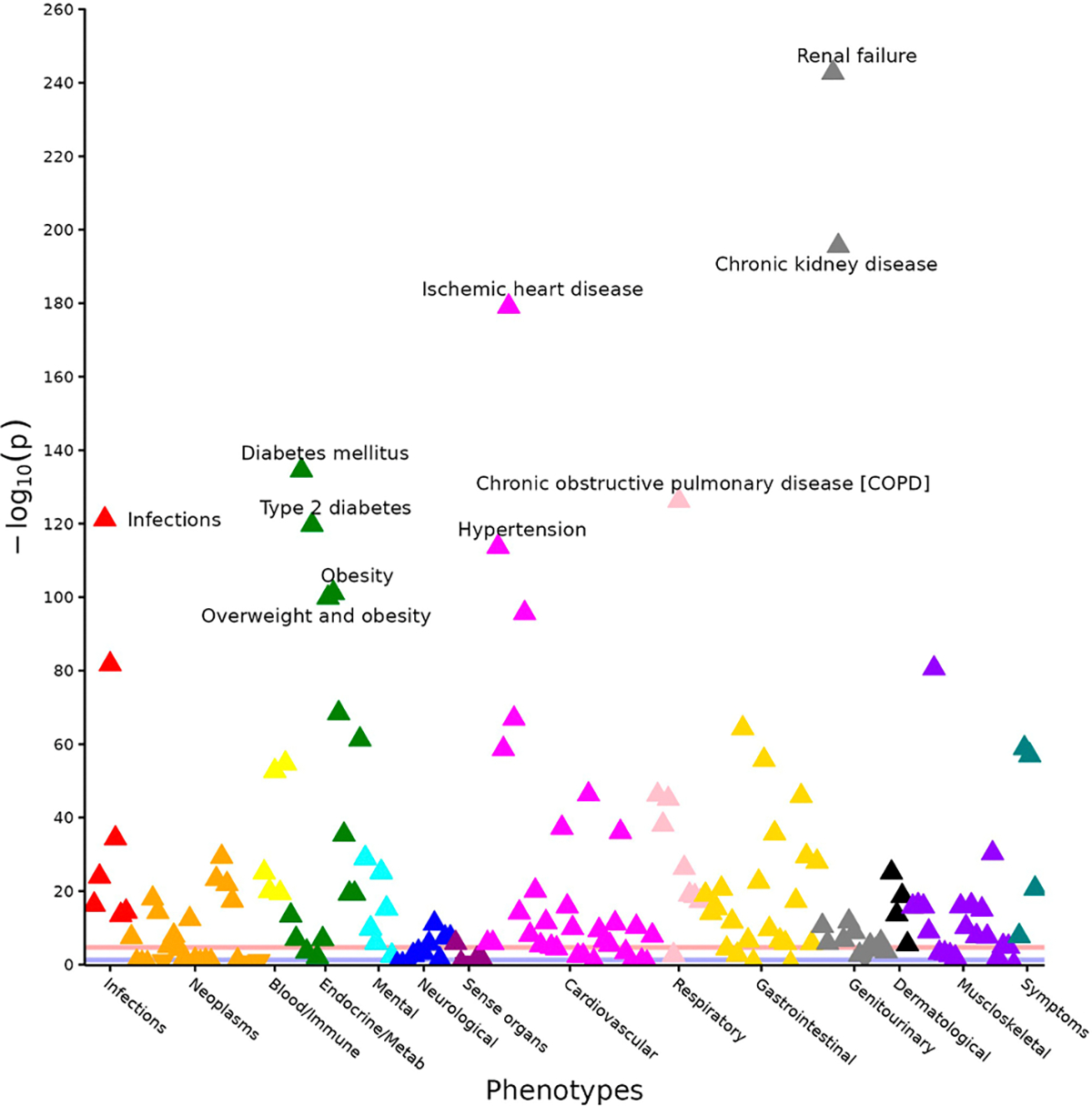
Phenome-wide association study of the proteomic risk score. Logistic regression models included covariates of age and sex with an outcome of severe diverticulitis. Annotations represent phecodes with the ten lowest *P* values. Horizontal red line reflects the Bonferroni-adjusted two-sided *P* value significance threshold (*P* < 2.1 × 10^‒5^).

In the PheWAS, the proteomic risk score was associated with greater odds of 125 conditions (Fig. 4). The three most strongly associated phecodes were “renal failure” (odds ratio [95% CI] 21.0 [17.6–25.1], *P* = 2.0 × 10^−243^), “chronic kidney disease” (OR [95% CI] 32.1 [25.6–40.4], *P* = 2.7 × 10^−196^), and “ischemic heart disease” (OR [95% CI] 6.0 [5.3–6.7], *P* = 8.3 × 10^−180^) (Supplementary Material). The most frequently represented systems among significant associations were cardiovascular (*n* = 24 phecodes), gastrointestinal (*n* = 18 phecodes), and musculoskeletal (*n* = 14 phecodes).

## Discussion

This study derived a plasma proteomic signature for severe diverticulitis and identified pathway-level disturbances in extracellular matrix and neuronal biology. Among participants without diverticular disease at study enrollment, a one SD increase in the proteomic risk score was associated with 48% greater risk of severe diverticulitis throughout follow-up. The proteomic risk score improved upon models with demographic and lifestyle factors though with declining utility over time. A PheWAS demonstrated that proteomic risk for diverticulitis was related to other traits, most notably renal dysfunction and cardiometabolic disease. Overall, this multiomic study suggests that for diverticulitis, genetic, and proteomic factors may provide insight into disease biology and complement clinical factors for individualized risk stratification.

Our results support distinct proteomic profiles among patients with severe diverticulitis. Prior genome-wide association studies have directed attention to connective tissue aberrations and gut neuronal biology^7,30–32^; however, none of associated genes in these studies had available plasma protein levels in our cohort. Despite this, our enrichment analysis replicated many similar pathways such as extracellular matrix remodeling and the neuronal system. Typically, mechanistic studies using distinct omics levels (i.e., genetic, transcriptomic, proteomic) focus on the tissues or cells involved in disease.^33^ While informative for capturing the cellular environment in disease states, the requirement of an invasive biopsy may limit clinical applications. Thus, our finding that unique proteomic signatures exist, and that these signatures may be detectable in plasma samples, has important implications for the feasibility of future translation to the clinical setting.

Plasma proteomic profiles were associated with the onset of severe diverticulitis. Our base model consisted of demographic and lifestyle factors known to be important: age, sex, body mass index, smoking, and dietary patterns.^1^ Relative to these factors, inclusion of the proteomic risk score and a polygenic risk score improved discrimination in training and testing sets. In our evaluation of three follow-up periods (2 y, 5 y, 10 y), performance of the protein risk model declined over time. As plasma proteomic states fluctuate,^34^ predictive utility may be limited to a specific time frame after sample measurement. Thus, future investigations of proteomic profiles should probe for time-dependent changes in performance and specify a follow-up time boundary beyond which the information is less likely to be useful. In general, this contrasts with heritable genetic variation which persists over time. As personalized molecular data become more robust, additional work is needed to optimize strategies of combining omics levels with distinct temporal characteristics.

We found that higher proteomic risk for diverticulitis was associated with greater odds of renal dysfunction^35–37^ and cardiometabolic conditions.^38^ Previously hypothesized roles for shared biology with these conditions include the disruption of colonic microflora and immune function for kidney disease, and accumulation of visceral adipose tissue with inflammatory dysregulation for cardiometabolic traits. In our study, the strongest association occurred with renal failure, which likely captures a signal from the renal transplant population. Immunocompromised patients are often considered to be a unique subgroup warranting independent consideration for management due to higher rates of complicated disease, failure of medical management, and morbidity after operative intervention.^3,39^ Given our inability to discern temporality with a PheWAS, we cannot confirm whether identified associations act as risk factors or sequelae of the diverticulitis episodes. However, further study of these PheWAS-identified associations may shed light on additional subgroups where distinct management algorithms may be warranted.

Clinical applications of multiomic diverticulitis signatures include shared decision-making in elective operative intervention and tailoring upstream lifestyle interventions to an at-risk subgroup. In oncologic diseases, tumor subtyping through gene expression patterns have become a routine adjunct for treatment decisions,^40^ while polygenic risk reports have improved screening in randomized trials.^41^ For diverticulitis, practice patterns in patient selection for elective sigmoid colectomy remain variable across centers.^42^ While prior guidelines relied on the number of episodes, the current focus on individualized circumstances has not been accompanied by improved stratification tools.^3^ Prior surveys of colorectal surgeons have demonstrated interest in omics-based risk reports specifically for recurrent uncomplicated diverticulitis.^8^ Our study supports existing conclusions about potential benefit for a diverticulitis polygenic risk score,^8,9^ while also suggesting a complementary role for proteomic signatures. Further, although genetic profiles are not modifiable, integrated molecular risk information may enhance focus on modifiable lifestyle components relevant to diverticulitis such as dietary patterns. From a preventative lens, identifying at-risk groups may motivate upstream behavioral modifications prior to the onset of a severe episode.^43^ Overall, a future risk report integrating clinical factors with molecular phenotypes may allow for greater individualization of treatment options for patients with diverticulitis. Prior to clinical implementation, future work is needed to establish best practices for presenting multiomic reports to providers and patients, to evaluate cost-effectiveness, and to externally validate the findings of the current study.

There are many limitations to this study. First, the cohort was limited to the UK Biobank. While an independent testing set was used when evaluating the performance of the proteomic risk score, additional studies in other biobanks are needed to externally validate these findings. This volunteer-based cohort is comprised primarily of healthy European participants, which may not fully represent other hospital-based populations. Second, we defined a construct of severe diverticulitis based on diverticular disease codes associated with inpatient admissions or operative procedures, and we are unable to confirm the distinction of complicated *versus* uncomplicated diverticulitis given known limitations in code-based phenotyping.^44^ Third, the UK Biobank selected a subset of plasma proteins for measurement based on roles in general disease without specific attention to diverticulitis. Thus, there may be plasma proteins that are relevant for a diverticular disease signature but unmeasured in the current cohort. Fourth, we used LASSO-based methods for selection and weighting of candidates included in the proteomic risk score, but this may fail to account for complex interactions between environment exposures and genetic patterns in score derivation. Fifth, the normalized protein expression used by Olink is a relative expression value, and future studies quantifying absolute values are needed prior to adoption in the clinical setting. Sixth, this study used a static snapshot of proteomic profiles at a single point in time for all participants. Future designs that incorporate repeated measurements would provide a more comprehensive representation of diverticulitis-associated proteins over time.

## Conclusions

Plasma proteomic profiles complemented genetic and lifestyle factors in risk stratification for diverticulitis. Future risk reports integrating clinical factors with molecular signatures may allow for greater personalization of treatment options and disease trajectories.

## Supplementary Material

Supplementary Material

Supplementary data related to this article can be found at https://doi.org/10.1016/j.jss.2025.06.093.

## Figures and Tables

**Table 1 –:** Cohort characteristics.

Variable	Training set (75%)		Testing set (25%)	
	Control N = 32,225	Severe Diverticulitis N = 429	Control N = 10,763	Severe diverticulitis N = 122

Age	56 (8)	60 (7)	56 (8)	60 (7)
BMI (kg/m^2^)	27.3 (4.8)	28.6 (5.1)	27.4 (4.8)	28.3 (4.2)
Sex				
Female	17,443 (54%)	257 (60%)	5843 (54%)	74 (61%)
Male	14,782 (46%)	172 (40%)	4920 (46%)	48 (39%)
Smoking status				
Never	17,863 (56%)	204 (48%)	5921 (55%)	50 (41%)
Current or prior	14,205 (44%)	223 (52%)	4798 (45%)	71 (59%)
Healthy diet score	4.31 (1.67)	4.11 (1.76)	4.33 (1.65)	4.07 (1.79)

Values represent mean (SD) or *n* (%).

**Table 2 –:** Time-dependent AUC of Cox proportional hazard models in the testing set.

Time	Training set		Testing set			
	Base	Full	Base	Genetic*	Protein*	Full*

2 y	0.72 [0.65–0.78]	0.84 [0.78–0.89]	0.71	0.72	0.73	0.74
5 y	0.69 [0.64–0.73]	0.83 [0.79–0.86]	0.70	0.75	0.71	0.75
10 y	0.68 [0.65–0.72]	0.81 [0.79–0.84]	0.70	0.72	0.71	0.73

Values represent area under the receiver operating characteristics curve. 95% confidence intervals reported using resampling from 200 bootstrapped replicates.

Model covariates: Base model included age at recruitment, sex, smoking status, body mass index, and healthy diet score. Genetic model includes base model covariates plus a polygenic risk score. Protein model includes base model covariates plus the proteomic risk score. Full model includes base model covariates plus the polygenic risk score and protein risk score.

AUC = area under the receiver operating characteristics curve.

* Likelihood ratio test relative to base model. Genetic: *P* = 5.0 × 10^−5^. Protein: *P* = 2.2 × 10^−4^. Full: *P* = 2.7 × 10^−6^.
