# Supplementary material for: Plasma Proteomic Signatures for Diverticulitis Risk Stratification
Source: J Surg Res. Author manuscript; Available in PMC 2026 May 11. (PMC13159074; doi:10.1016/j.jss.2025.06.093)
Supplement: Supplementary Material [file NIHMS2161491-supplement-Supplementary_Material.docx]

## Supplementary Material

### Cohort Phenotyping

Diverticular disease diagnosis codes were obtained from International Classification of Diseases, 10^th^ revision (ICD-10) or 9^th^ revision (ICD-9) vocabularies. Codes included K57 for ICD-10 and 562 for ICD-9. Procedure codes were obtained from the Operating Procedure Codes Supplement version 4 (OPCS4) or version 3 (OPCS3) classification system. Codes for colectomy or drainage were H04, H05, H06, H07, H08, H09, H10, H11, H29, T34, T45, 402.1, 446, 460. Participants who subsequently withdrew from the study after enrollment were excluded from analysis. The duration of study follow-up was defined as the time from study enrollment to the date of final follow-up, which occurred with either the date of the first diverticulitis episode for cases or the date of last known follow-up for controls.

Counts of participants according to each inclusion criterion are shown in Supplementary Table 1, while the median (IQR) survival time for cases was 6.0 (5.7) years with a distribution shown in Supplementary Figure 1.

| Supplementary Table 1: Cohort phenotype criteria counts | |
| --- | --- |
| **Criteria** | **Count of participants** |
| Death due to diverticular disease | 10 |
| Colectomy or percutaneous drain for diverticular disease | 28 |
| Multiple inpatient admissions for diverticular disease | 513 |


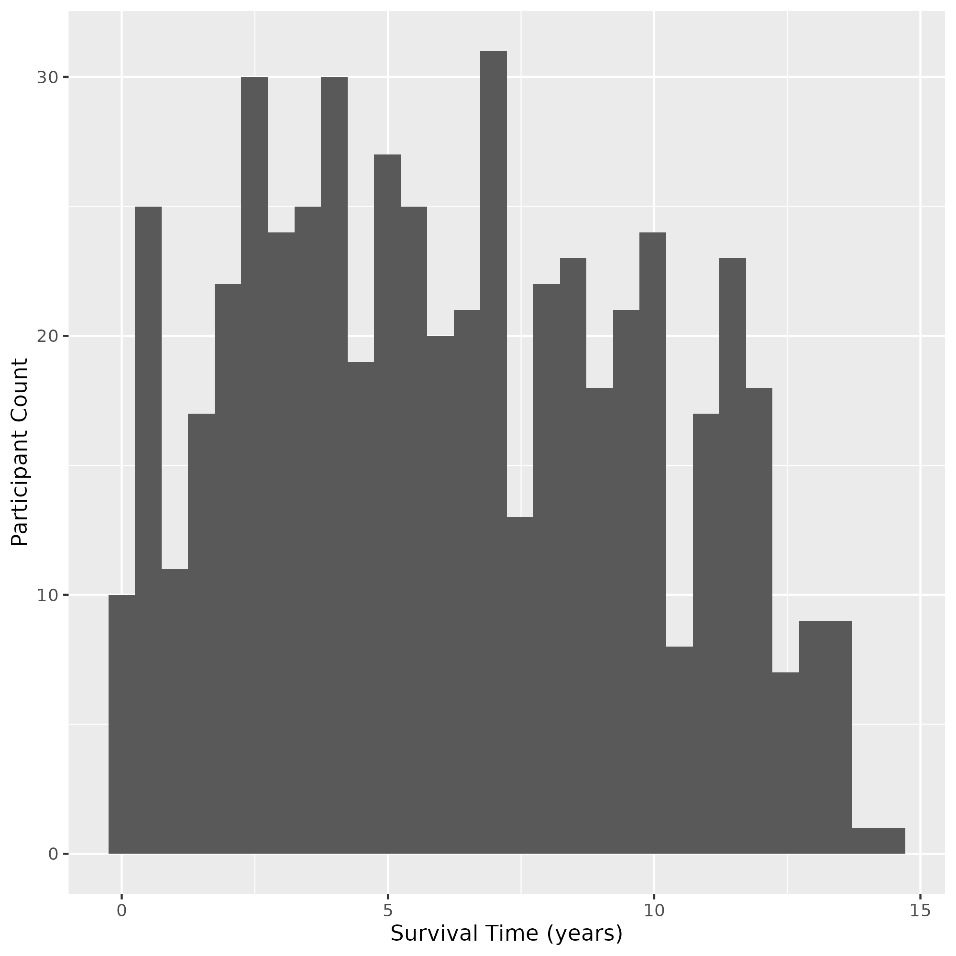


Supplementary Figure 1: Histogram of survival time among participants who developed severe diverticulitis throughout follow-up

### Model Covariates

#### Dietary Patterns

A healthy diet score was calculated based on concordance with national guidelines in consumption patterns of fruit, vegetable, fish, processed meat, red meat, whole grains, and refined grains that were self-reported at the enrollment visit (Supplementary Table 2). This was coded as either “Favorable” or “Unfavorable” using previously described criteria and the American Heart Association recommendations.^1,2^

| Supplementary Table 2: Defining a Health Diet Score | | |
| --- | --- | --- |
| **Pattern** | **Criteria for favorable** | **Scoring comments** |
| Fruit intake | ≥2.5 servings per day | If at least 4 of the 7 dietary components are favorable then diet score is "favorable" |
| Vegetable intake | ≥2 servings per day |  |
| Fish intake | ≥2x per week |  |
| Processed meat intake | ≤1x per week |  |
| Red meat intake | ≤2.5x per week |  |
| Whole grains | ≥3 servings per day |  |
| Refined grains | ≤1.5 servings per day |  |

#### Polygenic Risk Score

The polygenic risk score that was used in this study was derived from genome-wide association study summary statistics of diverticular disease from the FinnGen biobank, version R10.^3^ This score was optimized for the severe diverticulitis phenotype in Vanderbilt University Medical Center’s institutional biobank, BioVU.^4^ BioVU samples were sequenced on the Illumina Multi-Ethnic Genotyping Array and imputed through the TOPmed imputation server after initial quality control steps (minor allele frequency >1%, Hardy Weinberg Equilibrium exact test p-value threshold of 1 x 10^-10^, sex check, sample missingness <3%, sample heterozygosity within 3 standard deviations of the mean). Participants genetically similar to European-like reference samples were identified through principal components analysis on a subset of directly genotyped and Linkage Disequilibrium-pruned single nucleotide polymorphisms. Participants with no more than second degree relatedness were kept using PLINK2s implementation of the KING robust estimator. The PRS-CS software was used to calculate the polygenic risk score using four values for the hyperparameter phi (1 x 10^0^, 1 x 10^-2^, 1 x 10^-4^, 1 x 10^-6^). The phi value that maximized Nagelkerke’s R2 on the liability scale in a logistic regression model adjusting for age, sex at birth, body mass index, ever smoking status, and the first 10 genetic principal components was carried forward to the UK Biobank.

#### Proteomic Risk Score

The antibody-based Olink Explore 3072 PEA quantified plasma proteomic expression levels among a subset of 54,219 UK Biobank participants.^5^ We excluded proteins with more than 10% missingness in our cohort (n = 1,464 proteins excluded) and samples with more than 20% missingness across all proteins (n = 1,615 samples excluded). Remaining missing protein expression levels were imputed with the cohort median value. From the perspective of included proteins, samples in our cohort had a mean (SD) percent missingness of 2.2% (3.5%) (Supplementary Figure 2). From the perspective of samples, the mean (SD) percent missingness of proteins was 1.6% (0.01%) (Supplementary Figure 3).


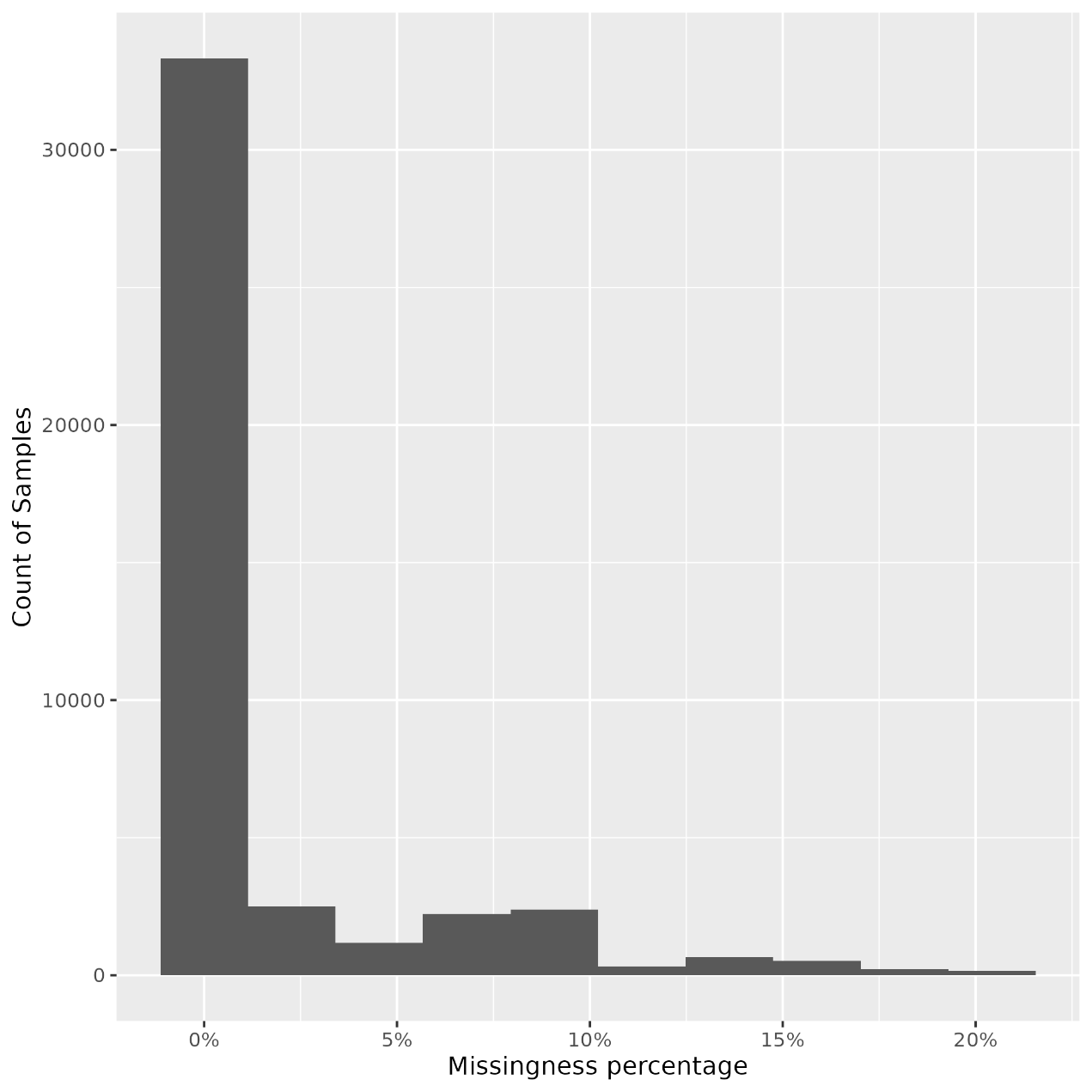


Supplementary Figure 2: Histogram of sample missingness percentage. Percent missingness for each sample in the cohort was calculated by dividing the number of proteins with missing expression values over the number of included proteins included.


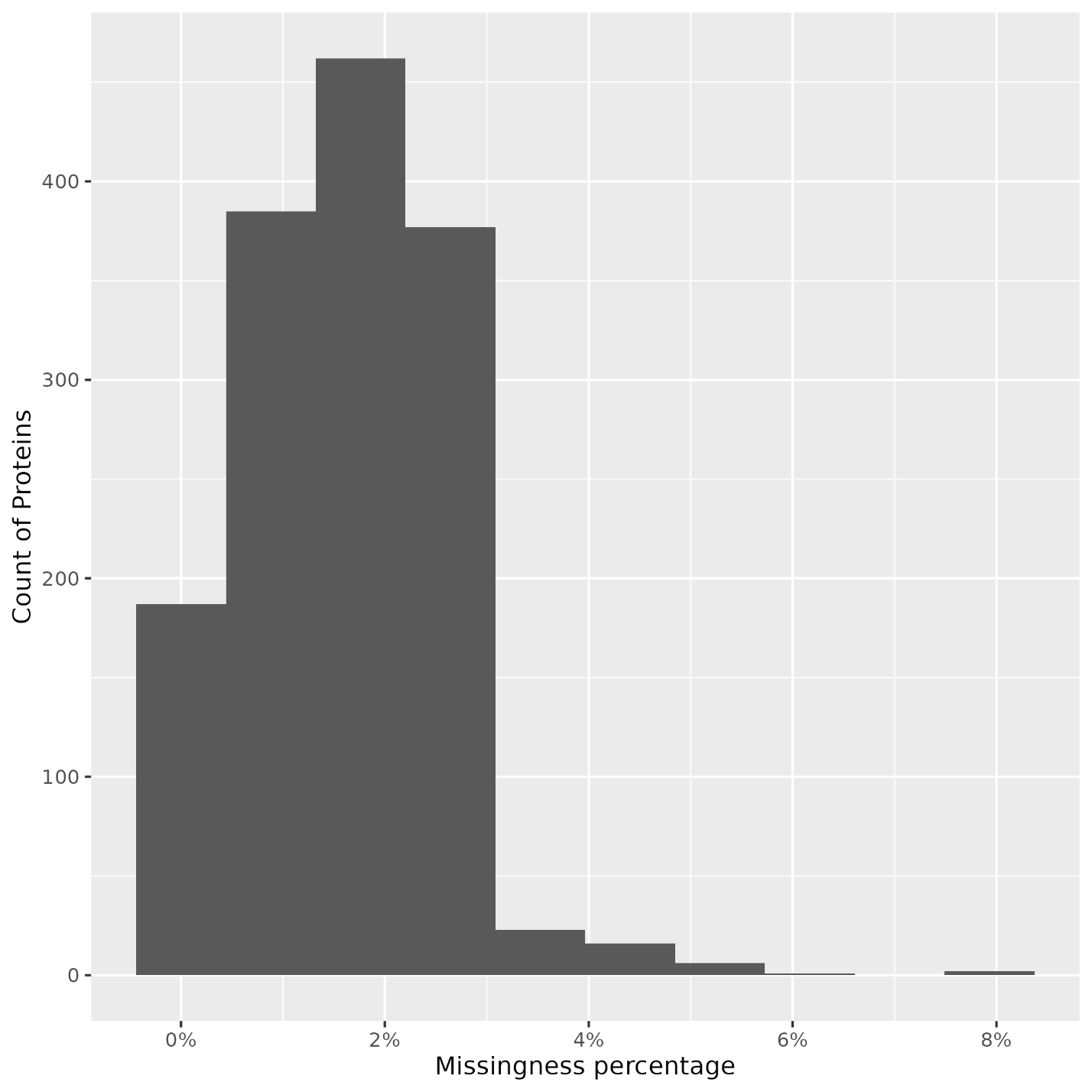


Supplementary Figure 3: Histogram of protein missingness percentage. Percent missingness for each protein was calculated by dividing the number of samples with a missing expression value by the number of samples in the cohort

To calculate the proteomic risk score, eligible samples from the training set were included in a Least Absolute Shrinkage and Selection Operator (LASSO) regression model adjusted for age and sex. The model outcome was severe diverticulitis (reference: controls without diverticular disease). Of the 151 retained proteins, the ten proteins with the largest LASSO coefficient magnitudes are shown in Supplementary Table 3. To calculate the proteomic risk score for a participant, the LASSO coefficient of each retained protein was multiplied by the protein expression value, then summed across all proteins. The score was standardized to a mean of 0 and standard deviation of 1 in the cohort. The distribution of the proteomic risk score when stratified by phenotype status is shown in Supplementary Figure 4.

| Supplementary Table 3: Ten proteins with largest weights in proteomic risk score | | | | |
| --- | --- | --- | --- | --- |
| **Protein abbreviation** | **Protein name** | **UniProt ID** | **Putative function** | **LASSO coefficient** |
| ctsv | Cathepsin L2 | O60911 | Cysteine protease | -0.109 |
| itga6 | Integrin alpha-6 | P23229 | Structural role in hemidesmosomes for epithelial cells | -0.075 |
| cntn1 | Contactin 1 | Q12860 | Mediates cell surface interactions during nervous system development | -0.070 |
| bmp6 | Bone morphogenetic protein 6 | P22004 | Essential role in cartilage and bone formation, iron metabolism | -0.069 |
| sh2d1a | SH2 domain-containing protein 1A | O60880 | Regulates receptors of the signaling lymphocytic activation molecule family | -0.068 |
| gga1 | ADP-ribosylation factor-binding protein GGA1 | Q9UJY5 | Trafficking between trans-Golgi network and endosomes | -0.066 |
| f9 | Coagulation factor IX | P00740 | Vitamin K-dependent plasma protein participating in intrinsic pathway of blood coagulation | 0.067 |
| ncs1 | Neuronal calcium sensor 1 | P62166 | Involved in long-term synaptic plasticity and neuron differentiation | 0.072 |
| agr2 | Anterior gradient protein 2 homolog | O95994 | Role in the production of mucus by intestinal cells | 0.084 |
| trem2 | Triggering receptor expressed on myeloid cells 2 | Q9NZC2 | Microglial activation, proliferation, migration, apoptosis, and expression of pro-inflammatory cytokines | 0.086 |
| LASSO: Least Absolute Shrinkage and Selection Operator. | | | | |


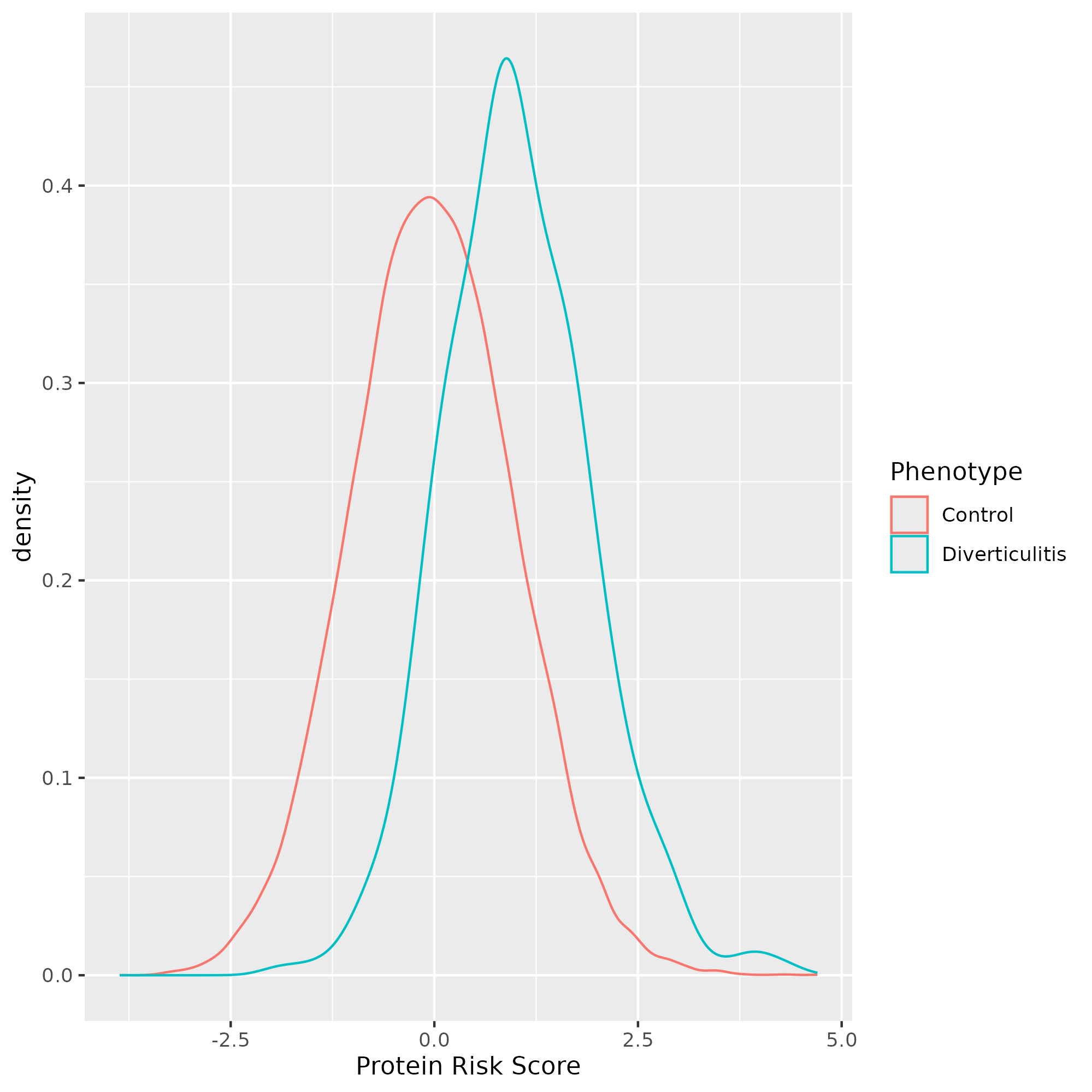


Supplementary Figure 4: Distribution of the proteomic risk score in cases of severe diverticulitis versus controls without diverticular disease

#### Variable Missingness

Supplementary Table 4 depicts the missingness for each variable included in the Cox proportional hazards model.

| Supplementary Table 4: Covariate Missingness | |
| --- | --- |
| **Variable** | **Number (percentage) missing** |
| Diet Score | 2,878 (6.6%) |
| Polygenic Risk Score | 411 (0.9%) |
| Body Mass Index (kg/m2) | 223 (0.5%) |
| Smoking | 204 (0.5%) |
| Age at Recruitment | 0 (0%) |
| Sex | 0 (0%) |

### Correlation analysis and phenome-wide association study

To assess whether the proteomic risk score captured information that was independent from the polygenic risk score, we computed Spearman’s rank correlation coefficient between the two variables (Supplementary Figure 5). The correlation was poor (Spearman’s ρ = 0.01). To assess whether the proteomic risk score was shared with other conditions, we performed a phenome-wide association study (PheWAS). The ten strongest associations are shown in Supplementary Table 5.


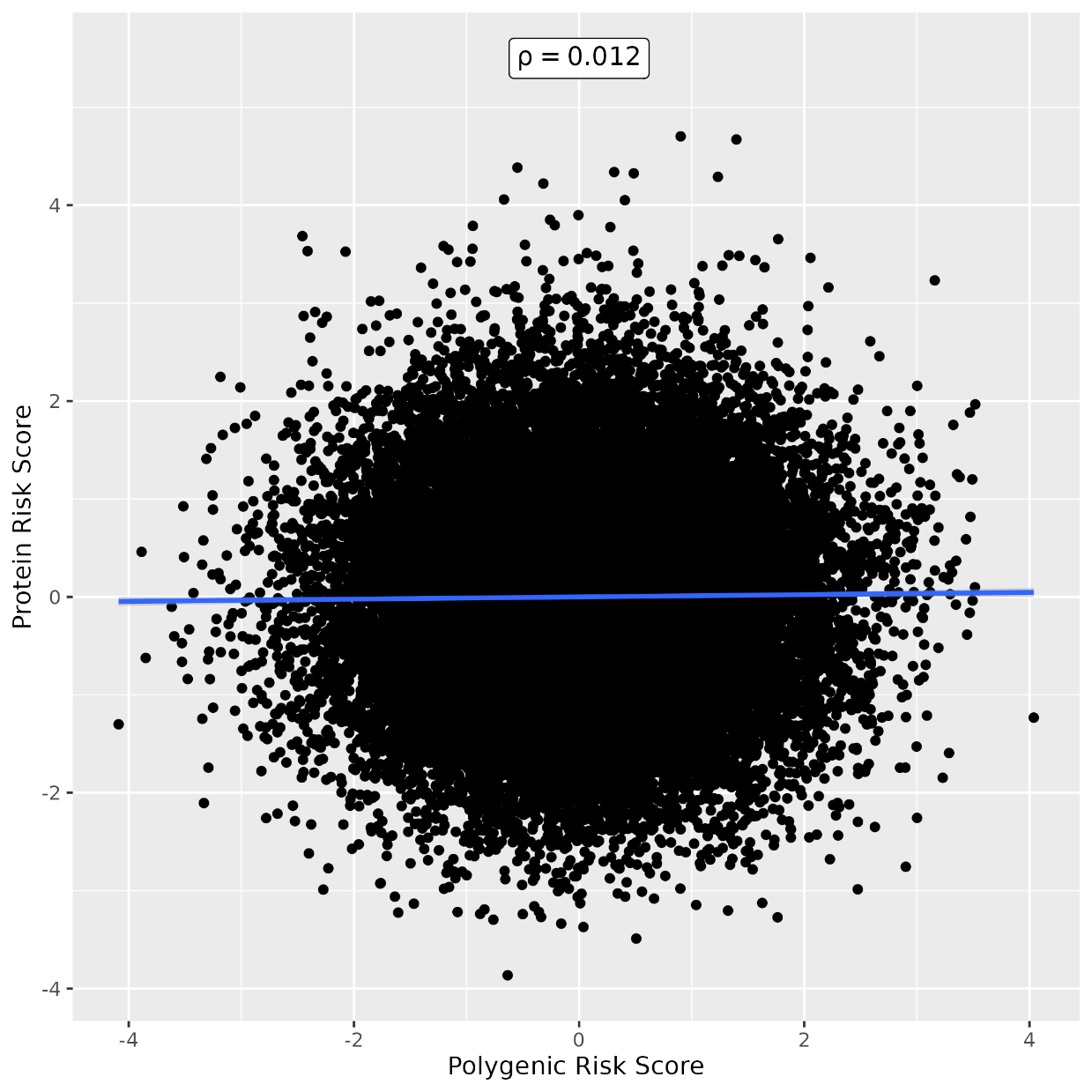


Supplemental Figure 5: Scatterplot and correlation between the proteomic risk score and the polygenic risk score. Rho represents Spearman’s rank correlation coefficient

| Supplementary Table 5: Top ten associations in phenome-wide association study of the proteomic risk score | | | | | | |
| --- | --- | --- | --- | --- | --- | --- |
| **Phenotype** | **Description** | **Odds Ratio (95% CI]** | **p-value** | **Total N** | **Number of cases** | **Number of controls** |
| GU_582 | Renal failure | 21 [17.6 - 25.1] | 1.956382e-243 | 40998 | 1357 | 39641 |
| GU_582.2 | Chronic kidney disease | 32.1 [25.6 - 40.4] | 2.728642e-196 | 41979 | 786 | 41193 |
| CV_404 | Ischemic heart disease | 6 [5.3 - 6.7] | 8.364648e-180 | 41828 | 3023 | 38805 |
| EM_202 | Diabetes mellitus | 14.1 [11.4 - 17.4] | 2.998310e-135 | 40563 | 858 | 39705 |
| RE_474 | Chronic obstructive pulmonary disease [COPD] | 14.1 [11.4 - 17.5] | 6.551918e-127 | 42170 | 794 | 41376 |
| ID_089 | Infections | 5.8 [5 - 6.7] | 7.116303e-122 | 40032 | 1781 | 38251 |
| EM_202.2 | Type 2 diabetes | 21.1 [16.4 - 27.3] | 2.222392e-120 | 40436 | 575 | 39861 |
| CV_401 | Hypertension | 37.8 [27.7 - 51.8] | 2.056042e-114 | 30378 | 383 | 29995 |
| EM_236.1 | Obesity | 40.8 [29.1 - 57.3] | 7.439089e-102 | 40602 | 277 | 40325 |
| EM_236 | Overweight and obesity | 38.8 [27.7 - 54.3] | 1.324133e-100 | 40600 | 281 | 40319 |

### References

1. Liu W, Wang T, Zhu M, Jin G. Healthy Diet, Polygenic Risk Score, and Upper Gastrointestinal Cancer Risk: A Prospective Study from UK Biobank. *Nutrients*. 2023;15(6):1344. doi:10.3390/nu15061344

2. American Heart Association. Healthy for Good: Healthy Living. www.heart.org. 2024. Accessed April 13, 2024. https://www.heart.org/en/healthy-living

3. Kurki MI, Karjalainen J, Palta P, et al. FinnGen provides genetic insights from a well-phenotyped isolated population. *Nature*. 2023;613(7944):508-518. doi:10.1038/s41586-022-05473-8

4. Roden DM, Pulley JM, Basford MA, et al. Development of a large-scale de-identified DNA biobank to enable personalized medicine. *Clin Pharmacol Ther*. 2008;84(3):362-369. doi:10.1038/clpt.2008.89

5. Sun BB, Chiou J, Traylor M, et al. Plasma proteomic associations with genetics and health in the UK Biobank. *Nature*. 2023;622(7982):329-338. doi:10.1038/s41586-023-06592-6
